# Carboxymethyl Chitosan Cinnamaldehyde Coated SilverNanocomposites for Antifungal Seed Priming in Wheat: A Dual-Action Approach Toward Sustainable Crop Protection

**DOI:** 10.3390/polym17152031

**Published:** 2025-07-25

**Authors:** María Mondéjar-López, María Paz García-Simarro, Lourdes Gómez-Gómez, Oussama Ahrazem, Enrique Niza

**Affiliations:** 1 Instituto Botánico, Departamento de Ciencia y Tecnología Agroforestal y Genética, Universidad de Castilla-La Mancha, Campus Universitario s/n, 02071 Albacete, Spain; maria.mondejar@uclm.es (M.M.-L.); mpaz.garcia3@alu.uclm.es (M.P.G.-S.); marialourdes.gomez@uclm.es (L.G.-G.); oussama.ahrazem@uclm.es (O.A.); 2 Facultad de Farmacia, Departamento de Ciencia y Tecnología Agroforestal y Genética, Universidad de Castilla-La Mancha, C/José María Sánchez Ibáñez s/n, 02008 Albacete, Spain; 3 Escuela Técnica Superior de Ingeniería Agronómica y de Montes y Biotecnología, Departamento de Ciencia y Tecnología Agroforestal y Genética, Universidad de Castilla-La Mancha, Campus Universitario s/n, 02071 Albacete, Spain

**Keywords:** seed priming, nanopriming, antifungal silver coated nanoparticles

## Abstract

Biogenic silver nanoparticles (AgNPs) were synthesized via a green chemistry strategy using wheat extract and subsequently functionalized with a carboxymethyl chitosan–cinnamaldehyde (CMC=CIN) conjugate through covalent imine bonding. The resulting nanohybrid (AgNP–CMC=CIN) was extensively characterized to confirm successful biofunctionalization: UV–Vis spectroscopy revealed characteristic cinnamaldehyde absorption peaks; ATR-FTIR spectra confirmed polymer–terpene bonding; and TEM analysis evidenced uniform nanoparticle morphology. Dynamic light scattering (DLS) measurements indicated an increase in hydrodynamic size upon coating (from 59.46 ± 12.63 nm to 110.17 ± 4.74 nm), while maintaining low polydispersity (PDI: 0.29 to 0.27) and stable surface charge (zeta potential ~ −30 mV), suggesting colloidal stability and homogeneous polymer encapsulation. Antifungal activity was evaluated against Fusarium oxysporum, Penicillium citrinum, Aspergillus niger, and Aspergillus brasiliensis. The minimum inhibitory concentration (MIC) against F. oxysporum was significantly reduced to 83 μg/mL with AgNP–CMC=CIN, compared to 708 μg/mL for uncoated AgNPs, and was comparable to the reference fungicide tebuconazole (52 μg/mL). Seed priming with AgNP–CMC=CIN led to improved germination (85%) and markedly reduced fungal colonization, while maintaining a favorable phytotoxicity profile. These findings highlight the potential of polysaccharide-terpene-functionalized biogenic AgNPs as a sustainable alternative to conventional fungicides, supporting their application in precision agriculture and integrated crop protection strategies.

## 1. Introduction

Cereal production remains a cornerstone of global food security, contributing as a fundamental component of human nutrition and as a primary input for livestock feed and industrial derivatives. Among cereals, wheat represents a major dietary staple, fulfilling approximately 40% of global bread consumption [1]. Despite its strategic importance, wheat yields are severely constrained by fungal pathogens, which are among the most destructive biological agents in agriculture. These phytopathogens are responsible for nearly 85% of crop disease losses, accounting for an estimated 20–40% reduction in annual global yields and inflicting economic damages that exceed USD 600 million to USD 4 billion annually [2,3].

To mitigate these losses, the agricultural sector extensively relies on fungicides; approximately 400,000 tons are applied globally each year, representing 20% of total pesticide usage [4]. However, conventional fungicide applications, particularly seed treatments and foliar sprays, often suffer from low specificity, poor retention, rapid degradation, and uncontrolled release. These shortcomings contribute to overapplication, environmental contamination, and declining efficacy due to the emergence of resistant fungal strains.

The widespread and prolonged use of synthetic agrochemicals has also raised substantial concerns regarding ecosystem health and food safety. Negative consequences include the suppression of beneficial soil microbiota, alterations in grain nutritional content, and the accumulation of toxic residues in food chains [5]. In response, regulatory frameworks are increasingly restrictive. Notably, the European Union’s Green Deal proposes a 50% reduction in pesticide use by 2030 and the progressive elimination of widely used chemical fungicides, such as triazoles [6].

Against this regulatory and ecological backdrop, nanotechnology has emerged as a promising platform for the development of next-generation plant protection products (PPPs). Among the various nanomaterials explored, silver nanoparticles (AgNPs) have received particular attention due to their broad-spectrum antimicrobial properties, low reactivity, and physicochemical stability [7,8]. AgNPs have demonstrated efficacy against diverse plant pathogens and have also shown potential in mitigating abiotic stresses, such as salinity, particularly in halotolerant crops like Pennisetum glaucum under 120–150 mM NaCl stress conditions [9]. Their unique properties make AgNPs viable alternatives to conventional PPPs, many of which are rapidly losing efficacy due to resistance development [10,11].

In parallel, plant-derived terpenes, especially cinnamaldehyde, a principal component of cinnamon essential oil, have shown potent antifungal, antibacterial, and antiviral activity against a wide range of phytopathogens. However, their high volatility, low aqueous solubility, and chemical instability under environmental stressors limit their standalone utility in agricultural formulations [12]. Recent advances in nanotechnology and polymer science have enabled the stabilization of such bioactives through encapsulation or conjugation with biocompatible matrices. In this context, biopolymers like chitosan and its water-soluble derivative, carboxymethyl chitosan (CMC), have attracted considerable interest due to their inherent antimicrobial activity, biodegradability, and functional versatility [13].

In our previous work, we demonstrated that CMC can be functionalized via imine (Schiff base) bond formation with cinnamaldehyde, yielding a polymer–terpene conjugate (CMC=CIN) that combines the physicochemical stability of the polymer with the antifungal potency of the bioactive [14]. This conjugate forms hydrogels with high water retention capacity and broad-spectrum antifungal efficacy, offering a promising foundation for the development of hybrid nanomaterials.

Building on this platform, the present study describes the synthesis of a bifunctional nanocomposite (AgNP–CMC=CIN) for application in sustainable crop protection. Silver nanoparticles were generated via a green chemistry route using wheat leaf extract as a biogenic reducing agent. Independently, CMC was conjugated with cinnamaldehyde and subsequently employed as a capping and stabilizing matrix for AgNPs. The resulting hybrid nanomaterial exhibited significantly enhanced antifungal activity compared to uncoated AgNPs, owing to the synergistic interaction between the metallic core and the bioactive polymer shell. Moreover, the formulation demonstrated a favorable phytotoxicity profile and improved seed vigor, indicating its potential as a next-generation nano-enabled seed priming agent in cereal crop protection programs, particularly under the imperatives of reduced agrochemical dependency.

## 2. Materials and Methods

### 2.1. Materials

Low-molecular-weight chitosan (CH) (50–190 kDa) with a 75–85% degree of deacetylation, 3-(4 5-dimethylthiazol-2-yl)-2 5-diphenyltetrazolium bromide (MTT), tebuconazole (TB), cinnamaldehyde (CIN), and all the solvents were supplied by Merck (Madrid, Spain). The fungal strains used in the antifungal bioassays included *Aspergillus niger* (ATCC 16888) and *Aspergillus brasiliensis* (ATCC 16404), sourced from the American Type Culture Collection (ATCC, Manassas, VA, USA). In addition, *Fusarium oxysporum* and *Penicillium citrinum* were kindly provided by the mycology collection reported in [15].

### 2.2. Synthesis of CMC

Carboxymethyl chitosan (CMC) was synthesized following a modified methodology based on the work in [16]. In a typical synthesis, 10 g of dry chitosan powder is suspended in 120 mL of isopropanol under vigorous stirring. Once a homogeneous suspension is achieved, 30 mL of 11.25 M NaOH solution (13.5 g NaOH) is added to the suspension to facilitate swelling and alkalization. This mixture is stirred for 1 h at 55 °C. Following alkalization, a solution of 15 g of monochloroacetic acid in 20 mL of isopropanol (at room temperature) is added dropwise to the reaction mixture, which is then stirred for an additional 30 min. The reaction is allowed to proceed for 4 h at 55 °C with continuous stirring. Upon completion of the reaction, 200 mL of ethanol is added to precipitate the synthesized CMC. The resulting suspension is then filtered using a Büchner funnel. The solid product is washed several times with ethanol to desalt and then dried overnight in an oven at 60 °C. The completion of the carboxymethylation reaction is monitored by two complementary indicators: (i) stabilization of the reaction mixture pH around neutrality (pH 7–7.5), indicating the consumption of sodium monochloroacetate; and (ii) disappearance of the characteristic acetyl stretching band near 1740 cm^−1^ in the FTIR spectrum, confirming successful substitution of hydroxyl groups on the chitosan backbone.

### 2.3. Synthesis of CMC=CIN and AgNP-CMC=CIN

#### 2.3.1. Synthesis of CMC=CIN

**Scheme 1:**
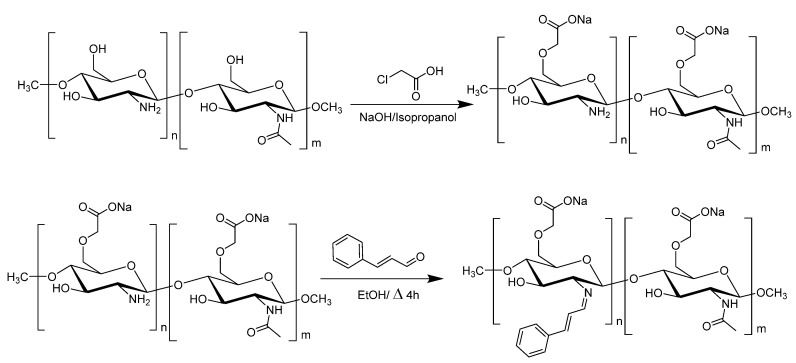
Synthetic procedure for CMC=CIN.

The synthesis of carboxymethyl chitosan–cinnamaldehyde Schiff base (CMC=CIN) was performed as illustrated in Scheme 1. Briefly, 1 g of CMC was dissolved in 100 mL of distilled water. This solution was then transferred to a round-bottom flask, submerged in an oil bath, and heated to 65 °C. Next, a solution of 3.65 mL of cinnamaldehyde (CIN) in 10 mL of absolute ethanol was added to the CMC solution [14,17]. The reaction mixture was maintained at 60 °C for 4 h under continuous stirring and reflux. Following the reaction, 200 mL of absolute ethanol was added with continuous stirring to induce the precipitation of CMC=CIN. The solid product was recovered by centrifugation at 9000 rpm for 5 min. To ensure the complete removal of unreacted cinnamaldehyde, the solid was subjected to a purification step: it was added to a 50:50 mixture of diethyl ether:ethanol and refluxed at 40 °C for 20 min. The solid was again recovered by centrifugation at 9000 rpm for 5 min and washed multiple times with the diethyl ether:ethanol mixture, with each wash followed by centrifugation. Finally, the purified solid was dried under a vacuum at 50 °C and stored at room temperature. The synthesized CMC=CIN conjugate was initially obtained as a dry solid after reaction and purification, ensuring the complete formation of Schiff base linkages and the removal of unreacted cinnamaldehyde. This solid intermediate was subsequently redissolved in distilled water to a defined concentration (2 mg/mL) prior to its use in the synthesis of AgNP–CMC=CIN

#### 2.3.2. Synthesis of Ag-CMC=CIN

Silver nanoparticles coated with CMC=CIN polymer were produced in two stages. In the first step, biogenic silver nanoparticles were synthesized using wheat leaf extract to which an equivalent volume of 25 mM AgNO3 was added. The nanoparticles were stirred and incubated for 1 h at 700 rpm [18]. Then, they were centrifuged for 15 min at 15,000 rpm and washed twice with ultrapure water. Nanoparticles were resuspended in an aqueous solution, and then, in the second step of the synthesis, AgNPs were added dropwise to a 2 mg/mL of CMC=CIN solution at a ratio of 6 mg of AgNPs per 16 mg of CMC=CIN. The mixture was then stirred for 5 h at room temperature in dark conditions at 300 rpm in order to carry out the coating process. Finally, the nanoparticles were centrifuged once again for 15 min at 15,000 rpm and washed twice to be resuspended in ultrapure water.

### 2.4. Instrumental Characterization of Ag-CMC=CIN

#### 2.4.1. UV-Spectrum of CMC=CIM and Ag-CMC=CIN

UV-vis spectroscopy was applied to evaluate the incorporation of CIN in a polysaccharide structure after a condensation reaction. The wavelength range went from 200 nm to 800 nm when observing the characteristic peaks that correspond to CIN presence. Briefly, CMC=CIN was completely dissolved in the aqueous solution, and then the sample was diluted several times to ensure the unsaturation of the absorbance to facilitate the spectra analysis.

#### 2.4.2. In Vitro Release Studies

To understand the impact of pH on the imine bond stability of water-soluble CMC=CIN, we quantified the amount of CIN released from the compound in PBS solutions at pH 5 and pH 7. The experimental setup involved dissolving 1 mg of CMC=CIN in 20 mL of PBS, ensuring uniformity with continuous stirring at 150 rpm. This solution was then encapsulated within a dialysis membrane (3500 Da cutoff) and immersed in another 20 mL of PBS, adjusted to the target pH values. The samples were incubated at 37 °C. Over time, 3 mL aliquots of the surrounding medium were withdrawn, and the concentration of CIN released was determined spectrophotometrically at 290 nm.

#### 2.4.3. DLS AgNP and AgNP-CMC=CIN

Particle size characterization of nano-formulations (size, zeta potential, and polydispersity index (PDI)) was determined by photon correlation spectroscopy through Dynamic light scattering (DLS) using a Zetasizer (3000HSM Malvern Ltd., IESMAT, Alcobendas, Spain) with the following specifications for AgNP-CMC=CIN: a chitosan refractive index (IR) of 1.700, absorption index of 0.010, and water solvent RI of 1.33, with a viscosity of 0.8872 cP. Measurements were performed in triplicate.

### 2.5. In Vitro Antifungal Activity of Ag-CMC=CIN

#### 2.5.1. Antifungal Effect in Spore Germination

The antifungal assay against the spores was performed according to with some modifications [19]. The pathogenic fungi *F. oxysporum*, *A. niger*, *A. brasiliensis*, and *P. citrinum* were collected in a spore suspension at a concentration of 3 × 10^4^ spores/mL (100 μL) and were transferred to a 96-well microtiter plate. The treatments were added by using the serial dilution method. Then, the plates were incubated for 48 h at room temperature, and all were treated with 10 μL of 3-(4 5-dimethylthiazol-2-yl)-2 5-diphenyltetrazolium bromide (MTT; 5 mg/mL in PBS; Sigma, Barcelona, Spain). Finally, plates were incubated overnight at room temperature, followed by the addition of 100 μL of MTT solvent (0.1 NHCl in anhydrous isopropyl alcohol) [20].

#### 2.5.2. Antifungal Evaluation of Nanoparticles in Infected Seeds

To assess the efficiency of new nanoparticles as effective seed nanopriming agents, we examined the germination rate post treatment. In brief, four 200 g batches of wheat (*Triticum vulgare*) seeds were divided into the following groups: control (-) (seeds neither inoculated with fungi nor treated), control (-) as the inoculated control (seeds inoculated with fungi but not treated), CMC=CIN (seeds inoculated with fungi and treated), AgNP, and AgNP-CMC=CIN (seeds inoculated with fungi and treated with both nanoparticles). In total, 200 g of seeds were soaked in the respective treatment at 5 mg/mL of each nanoparticle to ensure successful seed dressing and then dried at room temperature. The wheat seed batches were placed in individual pots for each treatment. Fungus-inoculated seeds were treated by adding suspensions of *F. oxysporum* with 3000 spores on the seed surface to ensure proper inoculation. The impact on seed germination was assessed by counting the number of surviving seeds. The experiment was conducted in triplicate. The plant development study was carried out over 126 days, and the surviving plants were collected for physiological and response analysis.

##### Assessment of Plant Growth Dynamics

Morphometric analyses were conducted at two time points. At 30 days after sowing, measurements were taken of both the shoot and root systems, including length and fresh weight. A second evaluation of the aerial part was performed at 126 days, recording its length and biomass accumulation again to assess long-term vegetative development.

##### Flavonoid, Polyphenol, Chlorophyll, Carotenoid, and Malondialdehyde Content in Treated Plants

The levels of polyphenols, flavonoids, and chlorophylls were analyzed to determine if the different treatments caused changes in cellular activity and plant physiology. Polyphenols and flavonoids are involved in defensive mechanisms against infection and environmental stress, so they were used to evaluate markers of plant antioxidant capacity and stress response. Also, the Folin–Ciocalteau method was used to quantify polyphenol content in the aqueous extract [21]. Briefly, 0.1 mL of aqueous extract was mixed with 2 mL of 2% Na_2_CO_3_, 2.8 mL of H_2_O, and 0.1 mL of Folin–Ciocalteau reagent. Color change was measured at 750 nm absorbance. Gallic acid was used as a standard at different concentrations (10–200 ppm). Also, a colorimetric method using AlCl_3_-6H_2_O was used to determine the flavonoid content of the aqueous extract. Briefly, 0.5 mL of the aqueous extract was mixed with 1.5 mL of ethanol, 0.1 mL of 10% AlCl_3_-6H_2_O, 0.1 mL of 1 M CH_3_COOK, and 2.8 mL of H_2_O. An evaluation of color change at 415 nm was performed after mixing. Quercetin (QE) was used as a standard at different concentrations (8–500 ppm). Finally, total chlorophyll content in the leaves of treated saplings was determined with 50 mg of powdered leaves and 300 µL of 80% acetone. The samples were then centrifuged at 10,000× *g* for 10 min, and supernatant absorbance was measured at 662 and 644 nm. Carotenoids were also measured at 450 nm. Lipid peroxidation was assessed through malondialdehyde quantification, following the protocol of Ref. [6]. Briefly, 0.3 g of fresh leaf tissue obtained from plants at 126 days was homogenized in 3 mL of 0.1% trichloroacetic acid (TCA) and centrifuged at 15,000× *g* for 15 min. A 1 mL aliquot of the supernatant was transferred to a test tube containing 3 mL of a 10% TCA solution with 0.65% thiobarbituric acid (TBA). The mixture was heated at 95 °C for 25 min and then rapidly cooled on ice to stop the reaction. Afterward, the samples were centrifuged at 10,000× *g* for 25 min. Absorbance was measured at 532 and 600 nm, using a blank solution containing equal volumes of TBA and TCA. The MDA concentration was calculated using the following equation:

$$MDA\;equivalent\;\left(\frac{mmol}{mL}\right)=\;\left[\frac{Abs\;532\;nm-Abs\;600\;nm}{155,000}\right]=10^{5}$$

### 2.6. Statistics

The obtained data were statistically analyzed using one-way ANOVA and Dunnet’s Multiple Comparisons test with the statistical software GraphPad Prism version 5.0.0 for Windows, GraphPad Software, San Diego, CA, USA. The differences were tested at <0.05 (95% probability level).

## 3. Results and Discussion

### 3.1. Spectroscopy Characterization of Ag-CMC=CIN

#### Uv-Vis Spectroscopy

Ultraviolet-visible (UV-Vis) spectroscopy is a widely utilized analytical technique in nanotechnology, particularly for evaluating the incorporation efficiency of bioactive compounds into carrier matrices and for characterizing surface modifications in functionalized nanoparticles. Its application has proven especially informative in systems involving chitosan and its derivatives grafted with essential oils or phenolic compounds [22].

**Figure 1:**
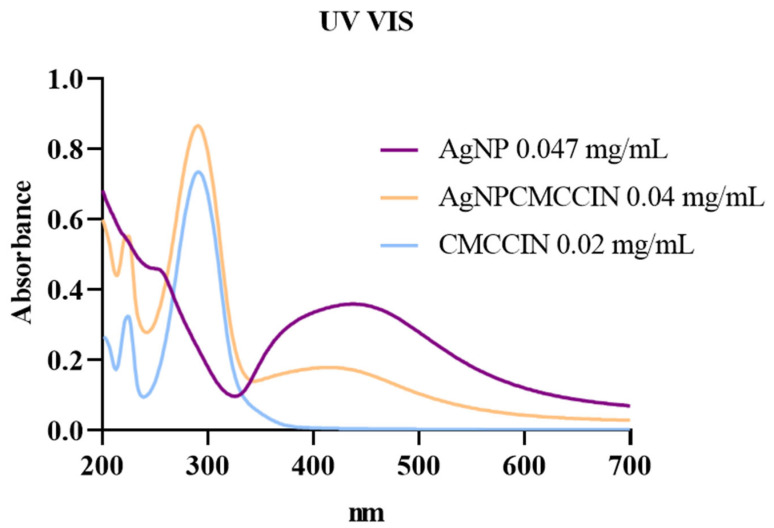
UV-VIS spectroscopy of biogenic nanoparticles, coated nanoparticles, and CMC=CIN.

Figure 1 presents the absorption spectra of carboxymethyl chitosan functionalized with cinnamaldehyde (CMC=CIN), biogenic silver nanoparticles (AgNPs), and the biofunctionalized complex AgNP-CMC=CIN within the spectral range of 200–700 nm. The reference spectrum for pure cinnamaldehyde (blue trace) exhibits a pronounced absorption band at 290 nm, consistent with the π→π* electronic transitions characteristic of this compound [23]. Notably, the spectrum corresponding to CMC=CIN (green trace) retains this distinctive absorption feature, suggesting the successful conjugation of cinnamaldehyde into the polysaccharide backbone, most likely through imine bond formation.

These findings are further supported by the work of [24], who reported analogous spectral signatures during the synthesis of a Schiff base derivative between hydroxypropyl chitosan and cinnamic acid. Their UV-Vis spectra revealed characteristic absorption maxima at 217 nm and 272 nm, affirming the presence of the aromatic system within the polymeric matrix.

Previous studies have examined absorbances in the UV-VIS spectrum of the CMC=CIN compound, comparing its spectrum with that of pure cinnamaldehyde and also with carboxymethyl chitosan [14]. Figure 1 shows the different spectra of biogenic AgNPs, the CMC=CIN polysaccharide, and the coated silver nanoparticles with the new CMC=CIN. Thus, the spectrum of silver nanoparticles shows a broad band with maximum absorption at 435 nm, whose shape matches the band indicated for a 1 h incubation period in the synthesis of silver nanoparticles, a time that coincides with the first step in the synthesis of our nanoparticles [25].

The spectrum obtained for the CMC=CIN compound shows a maximum absorption peak at 291 nm, characteristic of cinnamaldehyde, along with two other peaks of lower absorbance near 220 nm. Finally, the AgNP-CMC=CIN spectrum includes all the bands mentioned above, given that the broad band of AgNPs reappears between 400 and 500 nm, as well as the characteristic peaks of cinnamaldehyde, with one at 291 nm and two other peaks around 220 nm.

### 3.2. pH-Responsive Behavior of the Active Coating

**Figure 2:**
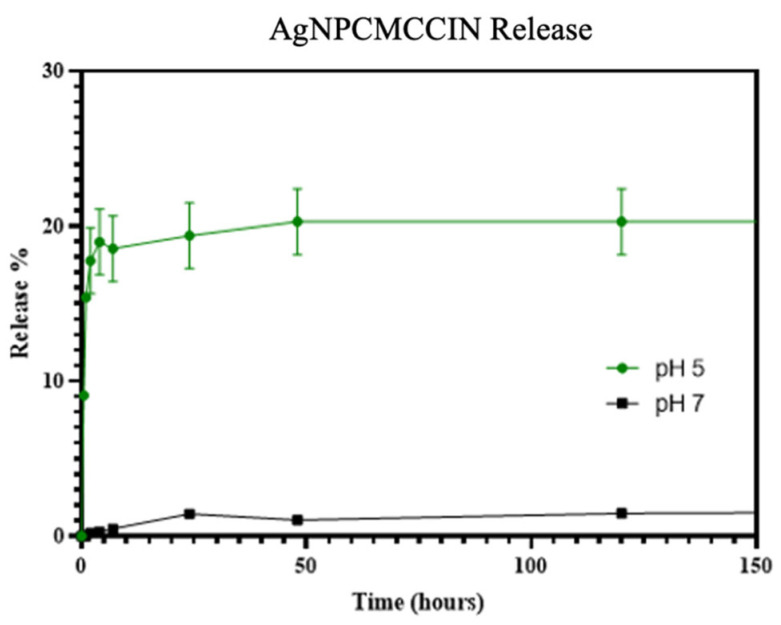
In vitro release of CIN from AgNPCMCCIN at pH 5 and pH 7.

AgNPCMCCIN showed remarkable stability at pH 7. There was practically no CIN release, with less than 2% of the total released after 15 days of continuous stirring at 37 °C, as illustrated in Figure 2. On the other hand, at a more acidic pH (pH 5), AgNPCMCCIN exhibited a rapid initial burst release within the first two hours but then remained stable for the subsequent 15 days. It is important to note that the release obtained was far from 100% despite the acidic environment, which confirms the improved stability of imide bonds. Another study examining Schiff base polysaccharide derivatives reported slightly higher degradation under acidic conditions, consistent with the increased stability conferred by the inclusion of the aryl group in the raw materials [26]. This means that Schiff base compounds are ideal platforms for controlled drug release into acidic environments [27].

### 3.3. Nanoparticle Characterization

Dynamic light scattering (DLS) was employed to determine the hydrodynamic diameter, polydispersity index (PDI), and zeta potential of the synthesized silver nanoparticles before and after functionalization. The corresponding results are summarized in Table 1.

The hydrodynamic radius of the uncoated biogenic silver nanoparticles (AgNPs), synthesized using wheat extract, was measured at 59.46 ± 12.63 nm. Following surface modification with carboxymethyl chitosan conjugated with cinnamaldehyde (CMC=CIN), the nanoparticle size increased to 110.17 ± 4.74 nm. This notable size increment confirms the successful deposition of the polymeric shell on the nanoparticle surface. Crucially, the PDI remained statistically unchanged (0.29 ± 0.01 vs. 0.27 ± 0.02), indicating that the coating process did not compromise the size homogeneity of the colloidal dispersion and suggesting effective stabilization by the CMC=CIN matrix.

Regarding zeta potential, both uncoated and coated nanoparticles exhibited comparable values (−29.03 ± 0.25 mV and −29.97 ± 2.99 mV, respectively), which are consistent with stable colloidal systems. These results suggest that the surface charge was not significantly altered by the coating, likely due to the consumption of free amino groups in chitosan during Schiff base formation with cinnamaldehyde. A ζ potential close to −30 mV is generally considered sufficient to prevent nanoparticle aggregation through electrostatic repulsion [28]. Although the absolute change in zeta potential values between uncapped and CMC=CIN-capped AgNPs appears modest, the increase from +17.2 mV (AgNPs) to +24.6 mV (AgNP–CMC=CIN) is nonetheless significant in colloidal terms. This elevation in surface charge is indicative of enhanced electrostatic repulsion between particles, which is known to mitigate aggregation and promote long-term colloidal stability in aqueous suspensions. The increase may be attributed to the presence of protonated amino and hydroxyl groups from the CMC backbone and residual cationic sites from CIN functionalization. Additionally, a higher positive surface charge can improve interactions with negatively charged fungal membranes, facilitating closer contact and more effective antimicrobial action. These findings are consistent with previously reported trends for polysaccharide-stabilized metallic nanoparticles [29,30].

These findings are in agreement with previous work by [31], who reported similar DLS profiles for silver nanoparticles synthesized via wheat extract. Interestingly, the particle size obtained after CMC=CIN coating in this study was smaller than those typically reported for silver nanocomposites synthesized by chemical reduction. For instance, ref. [32] reported particle sizes of 275 nm for chitosan nanoparticles and up to 373 nm for chitosan–silver nanocomposites.

### 3.4. Morphological and Size Evaluation of Nanoformulation by Electron Microscope

**Figure 3:**
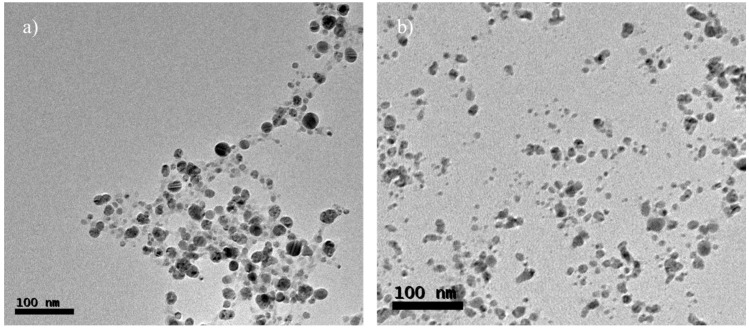
(**a**) AgNP and (**b**) AgNP-CMC=CIN.

Figure 3 presents transmission electron microscopy (TEM) images of silver nanoparticles (AgNPs) and (AgNP-CMC=CIN). In both cases, the nanoparticles exhibit predominantly spherical morphology with irregular shapes, characteristic of silver nanoparticles as previously reported [27].

However, when comparing the two samples, AgNP-CMC=CIN (Figure 3b) displays a more homogeneous and well-dispersed distribution, in contrast to the pronounced aggregation observed in the uncoated AgNPs. This stabilizing effect is attributed to the polymeric coating, which prevents excessive agglomeration through steric and/or electrostatic repulsion mechanisms. Similar stabilization phenomena have been documented in studies involving polymer or polysaccharide coatings such as alginate and chitosan [28].

Notably, no distinct polymer deposition is observable around the nanoparticles in the TEM images. This is primarily due to the high electron density of the silver core, which complicates direct visualization of the coating layer, a common challenge in such nanocomposite systems. Comparable observations were reported by [29] in silver nanocomposites stabilized with chitosan, where the coating was also not discernible by TEM, thus supporting the consistency of this interpretation. Furthermore, no differences in nanoparticle size were observed after coating with the new biopolymer, indicating that the coating does not alter the metallic structure of the nanoparticles, thus preventing their degradation. This distinction arises because dynamic light scattering (DLS) measures the hydrodynamic diameter of nanoparticles, which includes the hydration layer and any steric stabilization from surface coatings, whereas transmission electron microscopy (TEM) provides the physical core size of dehydrated particles under vacuum conditions. Therefore, slight discrepancies between DLS and TEM results are expected.

### 3.5. Evaluation of Antifungal Activity of CMC=CIN

#### 3.5.1. Increase in Antifungal Activity Nanoparticles by Active Coating

The minimum inhibitory concentrations (MICs) of the tested formulations varied depending on the fungal species and the antifungal agent employed. The results showed in Table 2 for *Fusarium oxysporum*, the lowest MIC was observed with tebuconazole (52 μg/mL), consistent with its established efficacy as a broad-spectrum triazole fungicide. Remarkably, the biofunctionalized AgNP-CMC=CIN nanoparticles demonstrated a MIC of 83 μg/mL, showing their potential as a competitive, eco-friendly alternative. In contrast, the uncoated biogenic AgNPs required a significantly higher MIC of 708 μg/mL, suggesting that functionalization with the CMC=CIN matrix markedly enhances antifungal activity.

In the case of *Penicillium citrinum*, *Aspergillus niger*, and *Aspergillus brasiliensis*, the MIC values for AgNP-CMC=CIN were comparable to those of tebuconazole and superior to both the uncoated AgNPs and the individual components (cinnamaldehyde and CMC=CIN). This behavior reinforces the hypothesis that synergistic interactions between the silver core and the bioactive coating potentiate the antifungal effects, particularly against filamentous fungi of agronomic relevance.

Previous studies support these findings. Ref. [32] reported a MIC of 100 μg/mL for chitosan–silver nanocomposites against *F. oxysporum*, which is notably higher than the 83 μg/mL achieved in the present study using biogenically synthesized AgNP-CMC=CIN. Similarly, ref. [33] described a MIC of 125 μg/mL for *A. niger* when treated with AgNPs biosynthesized using *Bacillus thuringiensis* cell-free extract, nearly six times higher than the concentration required by AgNP-CMC=CIN, further highlighting the superior efficacy of the functionalized formulation.

The enhanced antifungal activity of AgNP-CMC=CIN can be attributed to multiple well-documented mechanisms of action. Silver nanoparticles disrupt fungal viability through membrane potential destabilization, the inhibition of unsaturated fatty acid biosynthesis, the induction of mitochondrial dysfunction, and structural damage to the fungal cell membrane [34,35]. Moreover, phenolic compounds present on the surface of biogenic nanoparticles may further potentiate antifungal efficacy by interfering with mycelial development, particularly in species such as *A. niger*. This species is frequently found as a mycotoxigenic contaminant in agri-food matrices and serves as a model organism in industrial biotechnology, making it a relevant target for novel antifungal strategies [10].

Moreover, the enhanced biocidal activity observed in the AgNP–CMC=CIN composite compared to AgNPs alone can be attributed to a synergistic interaction between the silver nanoparticles and the cinnamaldehyde (CIN) moieties covalently bound to the carboxymethyl chitosan (CMC) matrix. While silver ions exert antimicrobial effects primarily via reactive oxygen species (ROS) generation and membrane disruption, cinnamaldehyde enhances this activity through its ability to destabilize fungal membrane integrity and interfere with enzymatic processes involved in respiration and energy metabolism. The polymeric scaffold, CMC, serves not only as a stabilizing agent but also as a functional delivery matrix that modulates release kinetics and promotes sustained activity. Such multi-target synergism is characteristic of hybrid nanosystems and has been reported in similar studies using chitosan–metal composites and plant-derived bioactives [36,37]. The integration of inorganic (Ag) and organic (CIN) biocidal agents within a biopolymeric carrier offers a promising strategy to enhance efficacy while reducing the total amount of silver required for antifungal action, thereby improving both performance and environmental compatibility.

#### 3.5.2. Successful Antifungal Activity of AgNP-CMC=CIN in Infected Seeds

To assess the efficacy of antifungal treatments during seed germination, both germination rates and visible signs of fungal mycelial growth were systematically recorded. At 48 h post sowing, germination rates were comparable between the negative control (non-infected) and *Fusarium oxysporum*-inoculated seeds primed with CMC=CIN, indicating no early-stage inhibition (Table 3). However, by day 5 (120 h), all control seeds had reached full germination, whereas CMC=CIN-treated seeds showed no further progression, suggesting an early stagnation in germination kinetics under fungal pressure despite initial resistance.

A similar pattern was observed for seeds treated with AgNP-CMC=CIN and inoculated with *F. oxysporum*, though with notable improvements. Specifically, AgNP-CMC=CIN-treated seeds exhibited a 15% higher germination rate compared to the infected control and demonstrated the lowest incidence of mycelial infection (2.5%). In contrast, the negative control (non-inoculated, untreated seeds) paradoxically presented visible mycelial growth on 23.3% of the seeds, possibly due to latent or environmental fungal contamination, pointing out the limitations of passive seed storage without protective treatment.

No statistically significant differences were found between seeds treated with biogenic AgNPs and those treated with the coated nanocomposite AgNP-CMC=CIN in terms of germination percentage. However, the coated nanoparticles consistently outperformed uncoated AgNPs in suppressing visible fungal growth, indicating a possible synergistic effect between silver and the cinnamaldehyde-functionalized CMC matrix.

Despite its relatively high germination rate, the CMC=CIN treatment was excluded from subsequent morphological and vegetative growth analyses due to its comparatively lower antifungal efficacy.

#### 3.5.3. Morphological Studies of Treated Plants

**Figure 4:**
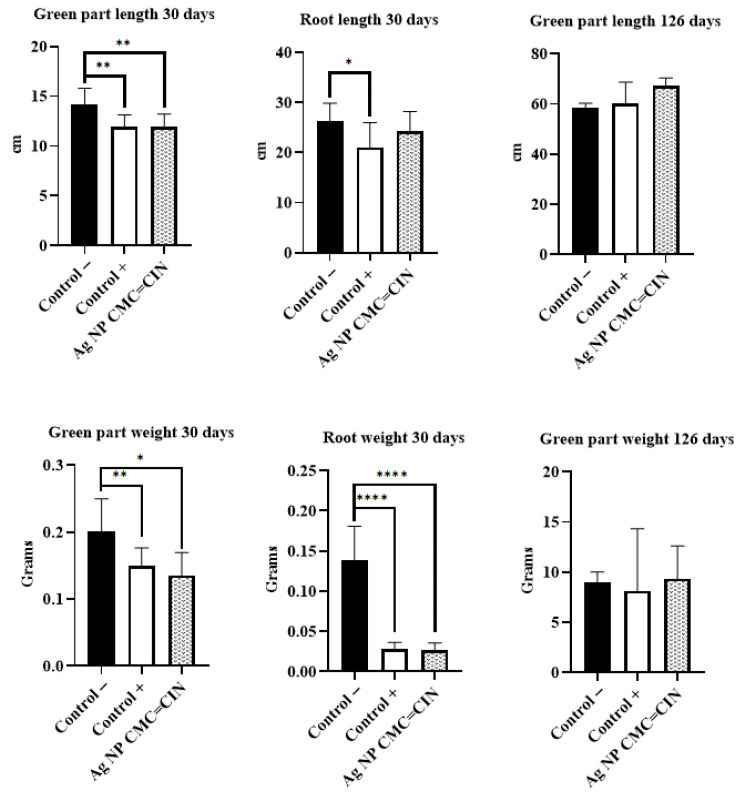
Morphological evaluation and statistical test at 30 days and 126 days of plants cycle. The result showed has been statistically evaluated using one-Way ANOVA with post hoc analysis (* *p* < 0.05, ** *p* < 0.01 and **** *p* < 0.0001.

**Figure 5:**
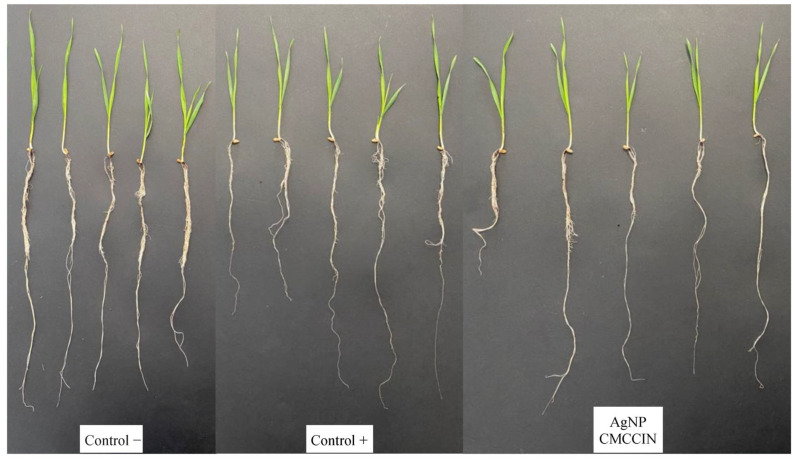
Appreciation of visual effects and changes in seedlings compared to controls and AgNP-CMC=CIN treatment.

Morphological evaluations conducted at 30 days post sowing revealed no substantial differences in shoot growth parameters between AgNP-CMC=CIN-treated plants and the positive control (Figure 4 and Figure 5). As expected, the negative control (non-inoculated and untreated) consistently exhibited superior performance in both aerial biomass and shoot length. However, a closer analysis of root development parameters indicated that seeds primed with AgNP-CMC=CIN achieved root lengths approaching those of the negative control, despite displaying lower root mass and density. This suggests that the nanocomposite treatment may facilitate root elongation during early vegetative stages, potentially enhancing initial soil exploration and nutrient acquisition without compromising root system architecture.

**Figure 6:**
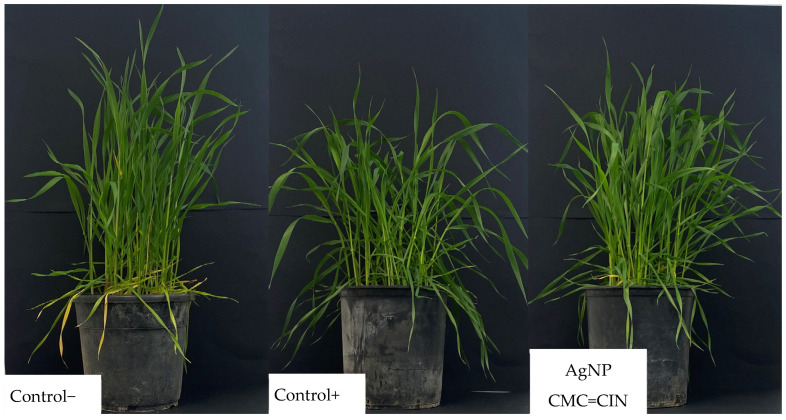
View of wheat pots 126 days prior to their morphological analysis and physiological parameters.

**Figure 7:**
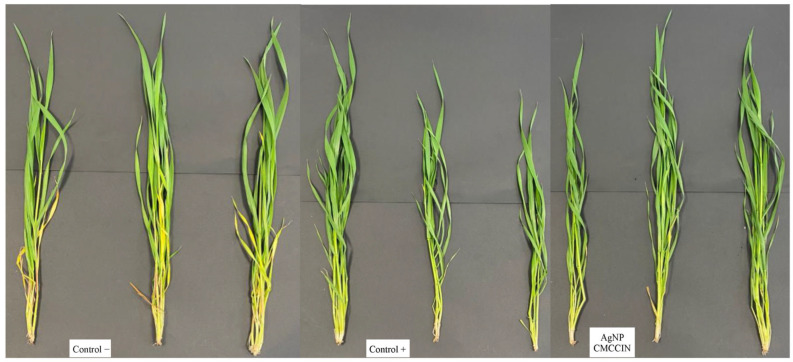
Morphological examination of the aerial part of wheat plants.

**Figure 8:**
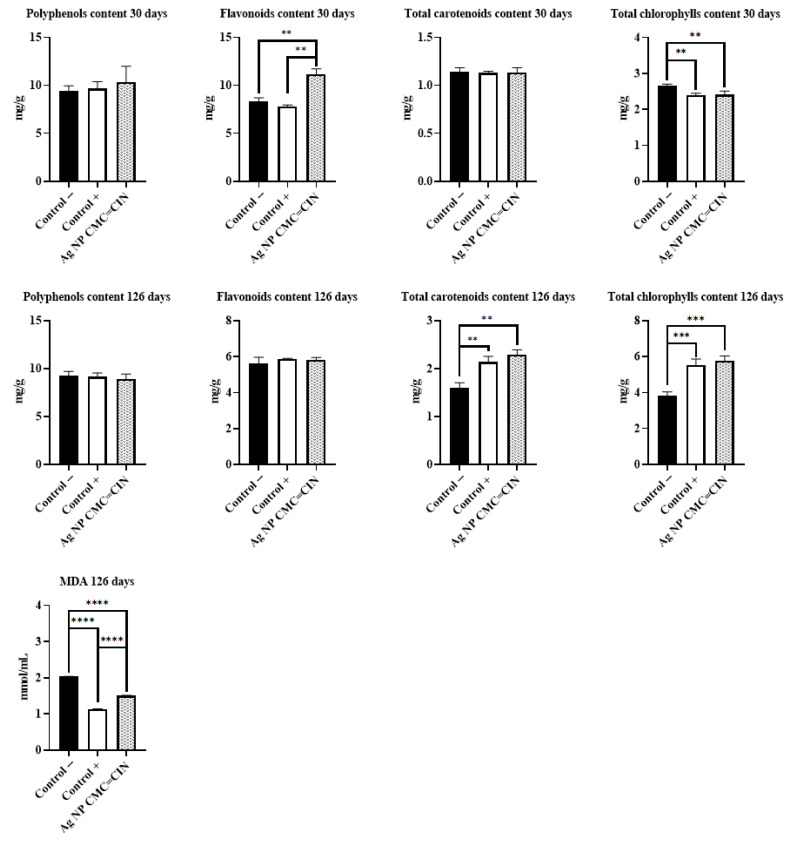
Physiological parameters and statistical test at 30 days and 126 days. The result showed has been statistically evaluated using one-Way ANOVA with post hoc analysis (** *p* < 0.01, *** *p* < 0.001 and **** *p* < 0.0001.

By day 126 (Figure 6, Figure 7 and Figure 8), more definitive trends emerged regarding the priming effect of AgNP-CMC=CIN. Plants derived from treated seeds exhibited a modest but reproducible increase in green shoot length relative to both positive and negative controls, which showed comparable and lower values (Figure 4). These results point to a delayed but sustained biostimulant effect, potentially attributable to the gradual release of bioactive components from the nanocomposite. The enhancement in aerial growth observed under biotic stress conditions also highlights the dual role of AgNP-CMC=CIN as both an antifungal agent and a growth-promoting formulation, supporting its candidacy as a next-generation plant protection product for cereal crops.

#### 3.5.4. Physiological Evaluation in Plants: Flavonoid, Polyphenol, and Chlorophyll Content in Treated Plants

Physiological analyses revealed that seed priming with AgNP-CMC=CIN induced a moderate increase in total polyphenol content in plant tissues, reaching values of approximately 10 mg/g dry weight, slightly exceeding those observed in both the negative and positive controls. This increasing tendency was more marked in total flavonoid accumulation, where levels approached 11 mg/g dry weight and showed a statistically significant difference (*p* < 0.05) compared to controls. The enhancement of flavonoid biosynthesis may be linked to mild oxidative priming triggered by the nanoparticle formulation, potentially activating defense-related metabolic pathways. In contrast, no significant variations were detected in carotenoid content among treatments, suggesting that the pigment biosynthesis pathways associated with light harvesting and photoprotection remained stable. Interestingly, chlorophyll levels were slightly higher in the negative control compared to both the AgNP-CMC=CIN and the pathogen-inoculated controls. These data may reflect an exchange between chlorophyll stability and stress signaling under biotic challenge, where plants prioritize the activation of secondary metabolism (e.g., polyphenol and flavonoid synthesis) over the maintenance of maximal chlorophyll concentrations. The physiological parameters at 126 days indicated that there were no significant variations in polyphenol and flavonoid values, while the carotenoid values of the positive control and the nanoparticle treatment were higher than those of the negative control. The same pattern was observed for chlorophyll content. This could be due to the fact that stress in the early stages of the plant activates its immune system and, therefore, also activates the pigment synthesis pathway. Malondialdehyde levels indicated that lipid peroxidation occurred at higher levels in the negative control, while both the positive control and the nanoparticle treatment showed lower levels. These values are related to the fact that plants that did not receive any treatment, that is, the negative control, tolerate stress situations less poorly. However, both the positive control and the plants treated with nanoparticles can exhibit a higher level of tolerance to different types of stress, specifically water stress.

The use of silver nanoparticles (AgNPs) in agricultural systems has raised justified concerns regarding potential toxicity to beneficial soil microbiota and environmental persistence. However, the concentration of silver used in our formulation is significantly lower than thresholds commonly associated with ecotoxicological effects. Specifically, the AgNP–CMC=CIN composite was applied at a concentration of 5 mg/mL for seed priming, corresponding to an estimated surface deposition of ≤50 µg of silver per gram of seed, which is in line with previously reported safe application levels for seed treatments. Moreover, encapsulation within the CMC=CIN matrix is likely to reduce the uncontrolled release of silver ions, as supported by the low Ag^+^ solubilization measured in our release studies (<5 ppb). This biofunctionalized matrix may act as a barrier, modulating silver bioavailability and thus mitigating potential risks to the surrounding soil microbiota. These findings are in agreement with the recent literature emphasizing the importance of polymeric capping agents in reducing the environmental footprint of AgNPs in agro-technological applications [38,39,40]. Future work will further explore the long-term effects of this formulation on rhizosphere microbial dynamics and nutrient cycling.

## 4. Conclusions

This study demonstrates the efficacy of a seed priming strategy based on biogenic silver nanoparticles (AgNPs) coated with a carboxymethyl chitosan (CMC) matrix covalently functionalized with cinnamaldehyde (CIN). The resulting nanohybrid (AgNP-CMC=CIN) exhibited antifungal performance comparable to conventional synthetic fungicides, while maintaining phytocompatibility and, in some cases, enhancing the key morphological and physiological parameters of early plant development. These findings reflect the potential of nano-priming technologies as a sustainable and precision-oriented alternative to traditional phytosanitary practices, particularly in light of ongoing regulatory restrictions on conventional agrochemicals. Given the paradigm shift toward agroecological models and circular bioeconomy frameworks, the development of biodegradable, low-toxicity nanomaterials is increasingly relevant. However, the environmental fate, bioaccumulation potential, and ecotoxicological thresholds of metal-based nanoparticles remain insufficiently characterized. Distinctions between biogenic and chemically or physically synthesized AgNPs, especially in terms of reactivity, toxicity, and environmental persistence, must be clarified through systematic studies. In parallel, the implications for human health, particularly regarding chronic exposure via food chains or agricultural handling, require further elucidation to establish acceptable exposure limits. These concerns reinforce the need for robust regulatory frameworks that are responsive to scientific innovation, capable of evaluating nano-enabled plant protection products, and aligned with the objectives of sustainable agriculture. Future research should integrate advanced materials characterization, omics-based biological assessments, and life cycle analysis to accelerate the responsible translation of nanotechnology into agricultural systems.

## Figures and Tables

**Table 1:** Measurements for average size, PDI, and Z potential for AgNP and AgNP-CMC=CIN.

NP	Size (nm)	PDI	Zeta Potential
AgNP	59.46 ± 12.63	0.29 ± 0.01	−29.03 ± 0.25
AgNP-CMC=CIN	110.17 ± 4.74	0.27 ± 0.02	−29.97 ± 2.99

**Table 2:** Minimum inhibitory concentrations (MIC) of control (tebuconazole), biogenic AgNPs, biogenic AgNP-CMC=CIN, and respective controls.

	Fungi (3000 Spores)			
Germinated Spore Treatment	*Fusarium oxysporum*	*Penicillium citrinum*	*Aspergillus* *niger*	*Aspergillus brasilensis*
AgNP (μg/mL)	708	<18	71	71
CIN (μg/mL)	416.25	182.25	416.25	416.25
CMC=CIN (μg/mL)	159.25	273.25	387.03	364.34
AgNP-CMC=CIN (μg/mL)	83	<21	<21	<21
Tebuconazole (μg/mL)	52	<13	<13	<13

**Table 3:** Viability of priming effects on wheat seeds.

	Viability					
Seed Priming Before Inoculation	48 h		72 h		120 h	
	Germination Rate %	Fungal Infection %	Germination Rate %	Fungal Infection %	Germination Rate %	Fungal Infection %
Control−	96.7	10	100	16.7	100	23.3
Control+	70	100	70	100	70	100
CMC=CIN	96.7	3.3	96.7	3.3	96.7	6.7
AgNP	85.5	20	85.5	20	85.5	20
AgNP-CMC=CIN	85	2.5	85	2.5	85	2.5

## Data Availability

Data are contained within the article.
